# NFAT5 Deficiency Alleviates Formalin-Induced Inflammatory Pain Through mTOR

**DOI:** 10.3390/ijms22052587

**Published:** 2021-03-04

**Authors:** Do Hyeong Gwon, Song I. Kim, Seoung Hun Lee, Chan Noh, Yeojung Kim, Sangwon Yun, Won Hyung Lee, Jun Young Oh, Dong Woon Kim, Jinpyo Hong, Sun Yeul Lee

**Affiliations:** 1 Department of Medical Science, School of Medicine, Chungnam National University, Daejeon 35015, Korea; dohyeong171@gmail.com (D.H.G.); kthddl2295@gmail.com (S.I.K.); visnu528@cnu.ac.kr (D.W.K.); 2 Department of Anatomy, Brain Research Institute, School of Medicine, Chungnam National University, Daejeon 35015, Korea; 3 Department of Anesthesiology and Pain Medicine, School of Medicine, Chungnam National University, Daejeon 35015, Korea; bacchuslee@naver.com (S.H.L.); jyfchrh@cnuh.co.kr (C.H.); yeojung80@naver.com (Y.K.); yfreedom03@naver.com (S.Y.); whlee@cnu.ac.kr (W.H.L.); 4 Department of Neuroscience and Physiology and Dental Research Institute, School of Dentistry, Seoul National University, Seoul 08826, Korea; ojy852@snu.ac.kr

**Keywords:** inflammatory pain, NFAT5, mTOR, c-Fos, ERK, NR2B

## Abstract

Nuclear factor of activated T cells (NFAT5) is a well-known transcription factor that regulates the expression of genes involved in osmotic stress. However, the role of NFAT5 in inflammatory pain remains unknown. Here, we studied the function of NFAT5 in inflammatory pain using NFAT5-heterozygous (Het) mice. To study inflammatory pain, we injected 10 µL of 2% formalin into the right hind paws of mice and monitored pain behaviors, such as licking, lifting, and flinching, for 60 min. After the first 15 min (phase I), there were no significant differences in pain behaviors between wild-type (WT) and NFAT5-Het mice. However, from 15–60 min (phase II), NFAT5-Het mice displayed significantly fewer pain behaviors compared to WT mice. Further, the expression levels of inflammatory-pain-related factors, including c-Fos, phosphorylated extracellular signal-regulated kinase (p-ERK), and phosphorylated *n*-methyl-D-aspartate receptor subunit 2B (p-NR2B), were significantly elevated in the spinal dorsal neurons of formalin-treated WT mice but was not elevated in NFAT5-Het mice. Similarly, c-Fos, p-ERK, and p-NR2B levels were significantly higher in glutamate-treated PC12 neuronal cells but were not affected by Nfat5 silencing in glutamate-treated PC12 cells. Altogether, our findings suggest that NFAT5 deficiency may mitigate formalin-induced inflammatory pain by upregulating mammalian target of rapamycin (mTOR) expression and downregulating its downstream factors in spinal dorsal neurons. Therefore, NFAT5 is a potential therapeutic target for the treatment of inflammatory pain.

## 1. Introduction

Inflammatory pain is characterized by increased activity of primary afferent nerves (nociceptors), resulting in spontaneous pain, hyperalgesia, and allodynia. Increased activity of these nociceptors contributes to peripheral sensitization, which occurs when inflammatory mediators generate abnormal responses to normal stimuli [1]. Peripheral sensitization also refers to increased sensitivity to inflammation following tissue damage. Inflammatory pain is divided into two categories: acute and chronic [2,3]. Acute inflammation caused by trauma or disease is a natural physiological response to injury that activates nociceptors and evokes protective behaviors to prevent additional tissue damage and promote tissue repair [2]. In contrast, chronic inflammation causes persistent tissue damage and unwarranted pain due to continual local release of inflammatory mediators, including cytokines [1]. Formalin-induced acute inflammatory pain is a valid and reliable model in rodents [4,5]. In this model, formalin is administered under the skin of the plantar surfaces of the hind paws, and animals are monitored for pain behaviors, such as licking, lifting, and flinching. Inflammatory pain in this model occurs in two temporally distinct stages: phase I and phase II. Phase I starts immediately after formalin injection and lasts for the first 15 min, while phase II begins at 15 min and continues to 60 min after formalin injection [6]. Phase I involves the direct action of noxious chemical stimulation, whereas phase II is mediated by a combination of peripheral-input- and formalin-induced central sensitization [7,8,9,10]. Understanding the molecular signaling that underlies inflammatory pain in this model would contribute to the development of strategies to mitigate pain caused by acute inflammation. Currently, mitogen-activated protein kinases (MAPKs), *n*-methyl-D-aspartate receptor subunit 2B (NR2B), and c-Fos are known to initiate formalin-induced inflammatory pain [6,11,12,13]. For example, inhibition of p38 MAPK phosphorylation via pre-emptive treatment with endomorphins attenuates inflammatory pain by reducing the production of inflammatory cytokines in dorsal root ganglion (DRG) neurons [14]. Moreover, silencing of Nr2b relieves pain in rats with chronic inflammatory pain [15]. Formalin injection results in spontaneous pain behaviors and increases the expression of the mu-opioid receptor, c-Fos, and phosphorylated extracellular signal-regulated kinase (p-ERK1/2), which participates in MAPK signaling in the ipsilateral trigeminal ganglion (TG) [16].

Nuclear factor of activated T cells (NFAT5), also known as a tonicity-responsive enhancer binding protein (TonEBP), is a member of the Rel family of transcription factors, which includes nuclear factor kappa B (NF-kB) and NFAT1–4 [17]. Generally, when tissues such as the kidneys, skin, and eyes are exposed to osmotic stress, NFAT5 protects them from hypertonic stimulation [18]. Interestingly, although NFAT5 was initially identified as a key component of cellular osmotic homeostasis [17], it can also be stimulated by inflammation independent of osmotic stress. For example, NFAT5 induces the expression of proinflammatory cytokines in macrophages by activating Toll-like receptors (TLRs) and stimulating the Rel family, including NF-κB [19,20]. Several studies have demonstrated that NFAT5 regulates inflammation. Chronic arthritis and inflammation are suppressed by inhibition of NFAT5 [18], while melatonin inhibits sirtuin-1-dependent nicotinamide phosphoribosyltransferase (NAMPT) and NFAT5 signaling in chondrocytes to attenuate osteoarthritis [21]. Furthermore, NFAT5-deficient macrophages cause less severe inflammatory pathology than wild-type macrophages, demonstrating that NFAT5-dependent regulation of macrophages exacerbates joint inflammation [22]. Although the studies mentioned above show that NFAT5 is mostly involved in regulating inflammation, the role of NFAT5 in inflammatory pain has not exactly been evaluated. In previous studies, since complete loss of NFAT5, specifically deletion of exons 6 and 7, results in late gestational or perinatal lethality [23], NFAT5-Het mice have mostly been used for in vivo studies, such as hippocampal inflammation [24], hypothalamic inflammation [25], and rheumatoid arthritis [17] studies.

Mammalian target of rapamycin (mTOR) is a serine–threonine protein kinase that plays pivotal roles in cell proliferation and differentiation in the nervous system [26,27]. Furthermore, mTOR signaling is activated by a wide range of stimuli, including growth factors, cytokines, and amino acids. The activation of mTOR controls protein synthesis via sequential phosphorylation of its downstream effectors, such as eukaryotic translation initiation factor 4E binding protein 1 (EIF4EBP1) and ribosomal protein S6 kinase B1 (RPS6KB1). This mTOR signaling contributes to a variety of physiological and pathological pain states, such as cancer, inflammatory, and neuropathic pain. For example, inhibition of mTOR signaling ameliorates peripheral nerve injury-induced neuropathic pain in the insular cortex and cingulate cortex [26,27]. Moreover, formalin-induced hypersensitivity and neuronal hyperexcitability are mediated by mTOR signaling [28], while the phosphatidylinositol 3-kinase (PI3K)/protein kinase B (AKT)/mTOR pathway contributes to bone cancer pain [29]. Although numerous studies have demonstrated that NFAT5 and mTOR participate in several kinds of inflammation, the relationship between mTOR and NFAT5 in inflammatory pain has not yet been elucidated.

In this study, we examined the function of NFAT5 in formalin-induced inflammatory pain using NFAT5 heterzygous (Het) mice and found that NFAT5 regulated mTOR and its downstream factors c-Fos, ERK, and NR2B. These pain-related factors co-localized with neuronal cells in the spinal cord dorsal horn, suggesting that neuronal cells were sensitized by noxious stimuli and that pain-related factors were secreted from the neurons. We propose that NFAT5 controls the generation of inflammatory pain and is suitable for further investigation as a target for inflammatory pain intervention.

## 2. Results

### 2.1. Formalin-Induced Inflammatory Pain Is Decreased in NFAT5-Het Mice

**Figure 1:**
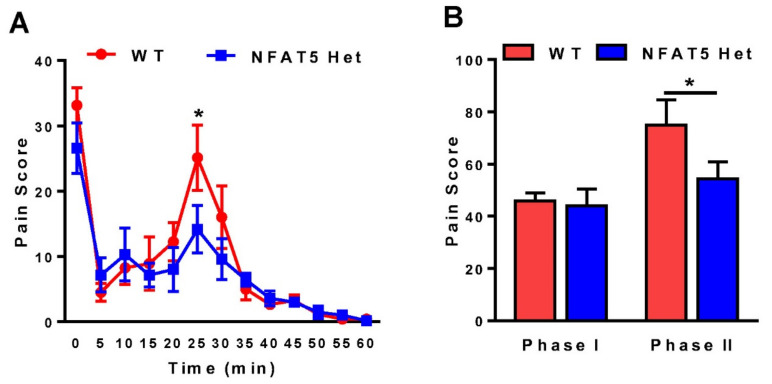
Formalin-induced pain behaviors in wild-type (WT) and NFAT5-Het mice. (**A**) After 10 μL of 2% formalin was administered under the skin of the plantar surfaces of the right hind paws in WT and NFAT5-Het mice, formalin-induced pain behaviors, including lifting, flinching, and licking, were measured for 60 min at 5 min intervals. Data are expressed as the means ± SEMs (two-way ANOVA with Bonferroni’s post-hoc test, * *p* < 0.05 vs. WT at 25 min, *n* = 8). (**B**) Total pain scores were classified as phase I (0–15 min) and phase II (15–60 min) in WT and NFAT5-Het mice. Data are expressed as the means ± SEMs (one-way ANOVA with Tukey’s post-hoc test, * *p* < 0.05 vs. WT, *n* = 7–8). ANOVA = analysis of variance.

Although NFAT5 plays a critical role in regulating the expression of genes involved in osmotic stress, its role in pain, including inflammatory pain, is not clearly understood. To test whether NFAT5 was engaged in inflammatory pain, we administered 10 µL of 2% formalin under the skin of the plantar surfaces of the right hind paws of wild-type (WT) and NFAFT5-Het mice to induce acute inflammatory pain. After administration of formalin, we observed pain behaviors, such as licking, lifting, and flinching of the affected paws, for 60 min at 5 min intervals (Figure 1A). Consistent with previous reports [6], mice showed typical patterns of pain behaviors during phase I (0–15 min) and phase II (15–60 min) (Figure 1B). Interestingly, we observed no behavioral differences between WT and NFAT5-Het mice during phase I, but we observed significant behavioral differences between the two groups during phase II, especially 25 min after formalin injection (Figure 1). These data indicate that NFAT5 may play a role in the second phase of inflammatory pain responses induced by formalin.

### 2.2. Activating Transcription Factor 3 Expression in DRG Neurons Is not Altered by NFAT5 Depletion in Formalin-Induced Inflammatory Pain

**Figure 2:**
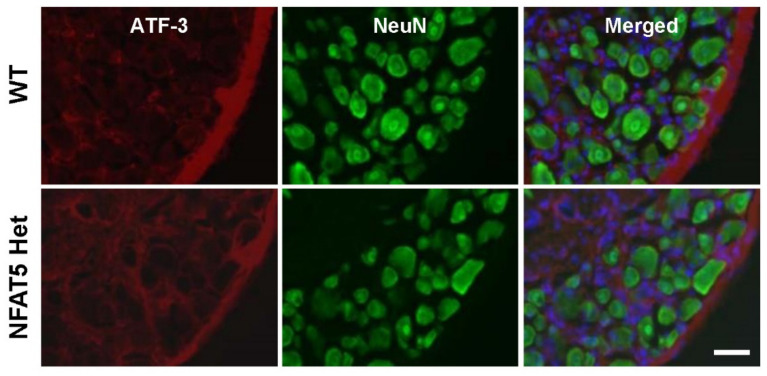
Formalin-induced ATF3 expression in dorsal root ganglia of WT and NFAT5-Het mice. At 1 h post-formalin injection, dorsal root ganglia (DRG) at lumbar (L) 3–5 from WT and NFAT5-Het mice were prepared and co-immunostained with anti-ATF3 (a neuronal injury marker) and anti-NeuN antibodies (a neuronal marker). Scale bar: 50 μm.

When sensory neurons are injured by physical or biochemical stresses, activating transcription factor 3 (ATF3) is upregulated in DRG neurons [30]. Based on the subdued pain response in formalin-treated NFAT5-Het mice, we investigated whether ATF3 expression in DRG neurons was altered by NFAT5 deficiency. We co-immunostained L5 DRG tissues from WT and NFAT5-Het mice 1 h post-formalin injection with anti-ATF3 and anti-neuronal nuclear protein (NeuN) antibodies. Surprisingly, there were no ATF3 immunoreactive (IR) neurons in either group (Figure 2), suggesting that the reduced acute inflammatory pain in formalin-treated NFAT5-Het mice is not influenced by ATF3 expression in DRG neurons.

### 2.3. c-Fos Expression Is not Induced by Formalin Administration in NFAT5-Het Mice

**Figure 3:**
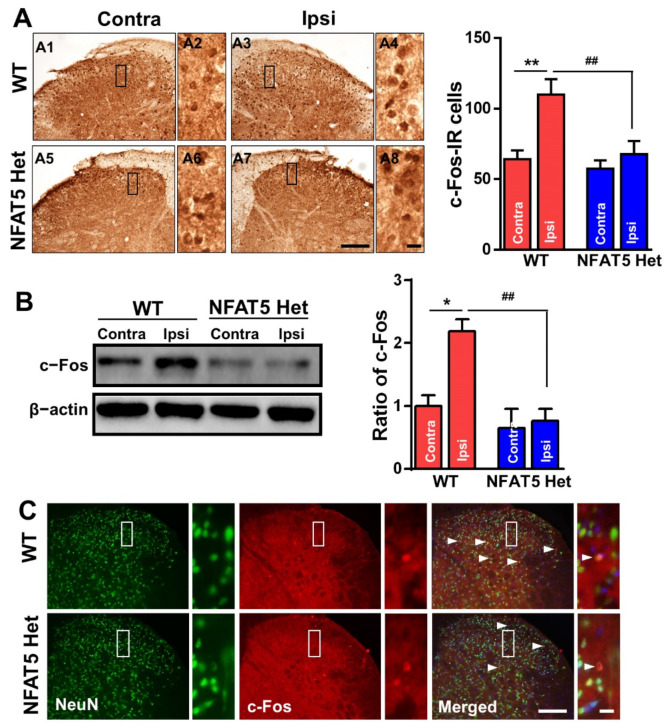
Formalin-induced c-Fos expression in the spinal dorsal horns of wild-type (WT) and NFAT5-Het mice. (**A**) At 1 h following formalin injection, lumbar (L) 4–6 fragments of the spinal cord from WT and NFAT5-Het mice were processed and immunostained with anti-c-Fos antibody. Scale bar: 50 μm (left), 20 μm (right). The numbers of c-Fos-immunoreactive (IR) cells were quantified in contralateral (contra) and ipsilateral (ipsi) sides of the spinal dorsal horn in WT and NFAT5-Het mice. Data are expressed as the means ± SEMs (one-way ANOVA with Tukey’s post, * *p* < 0.05 vs. WT contra, ** *p* < 0.01 vs. WT contra, ^##^ *p* < 0.01 vs. WT ipsi, *n* = 5). (**B**) The protein levels of c-Fos were assessed in contralateral and ipsilateral sides of the spinal dorsal horn 1 h after formalin injection by Western blot. Band intensities expressed as the ratio of c-Fos normalized to β-actin, which was used as a loading control. Data are expressed as the means ± SEMs (one-way ANOVA with Tukey’s post-hoc test, * *p* < 0.05 vs. WT contra, ^##^ *p* < 0.01 vs. WT ipsi, *n* = 5). (**C**) The same sections from (A) above were also co-stained with anti-c-Fos and anti-NeuN antibodies. Arrow heads indicate c-Fos/NeuN-positive cells. Scale bar: 50 μm (left), 20 μm (right).

As c-Fos contributes to pain transmission in formalin-induced inflammatory pain [6,16,31], we examined the levels of c-Fos in L4–6 spinal tissues from WT and NFAT5-Het mice 1 h post-formalin injection. In WT mice, we found that the number of c-Fos IR cells increased approximately 2-fold in the ipsilateral spinal dorsal horn compared to the contralateral spinal dorsal horn. In contrast, there was no difference in c-Fos immunoreactivity in the spinal dorsal horns of NFAT5-Het mice (Figure 3A). These observations were confirmed by Western blotting of spinal dorsal horn tissues from WT and NFAT5-Het mice 1 h after formal injection. In formalin-treated WT mice, c-Fos expression increased by approximately 2.2-fold in spinal dorsal horn lysates from the ipsilateral side compared to the contralateral side. Again, there was no difference in c-Fos expression in NFAT5-Het mice (Figure 3B). To examine c-Fos localization after formalin treatment, we co-immunostained spinal tissues with anti-c-Fos and anti-NeuN antibodies. NeuN is a marker of mature neurons, which are of interest in pain responses. Indeed, we found that c-Fos co-localized with NeuN-positive neurons in laminae 1 and 2 of the dorsal horn, and the number of c-Fos-positive neurons was greater in WT mice than in NFAT5-Het mice (Figure 3C). These data suggest that NFAT5 may contribute to formalin-induced inflammatory pain by regulating c-Fos expression specifically in neurons of the spinal dorsal horn.

### 2.4. ERK Is not Activated by Formalin Injection in NFAT5-Het Mice

**Figure 4:**
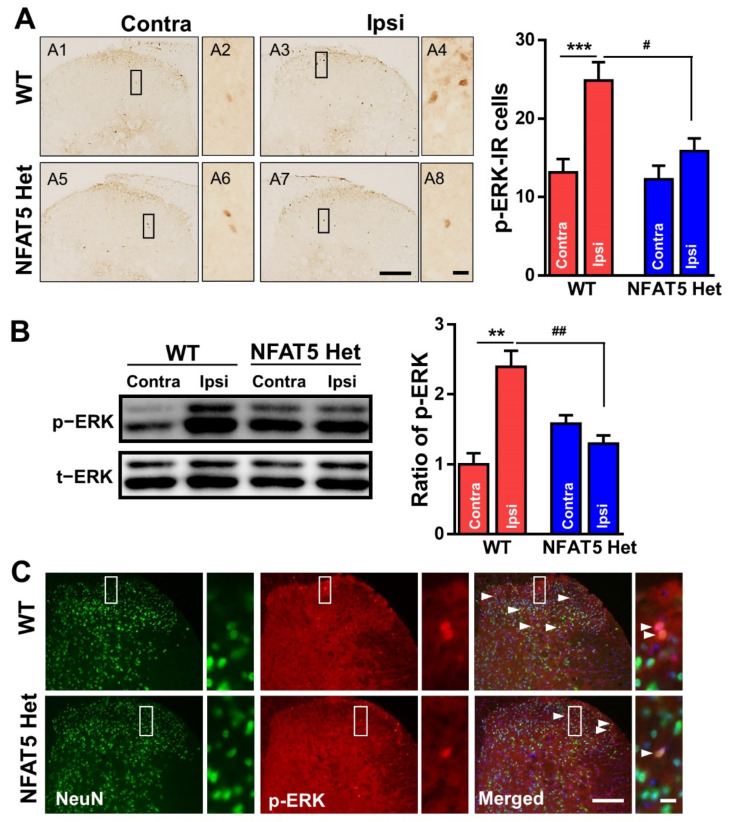
Formalin-induced phosphorylation of ERK in the spinal dorsal horns of WT and NFAT5-Het mice. (**A**) At 1 h post-formalin injection, the levels of ERK phosphorylation in the spinal dorsal horns of WT and NFAT5-Het mice were assessed by immunostaining with anti-p-ERK antibody. Scale bar: 50 μm (left), 20 μm (right). The numbers of p-ERK-immunoreactive (IR) cells were quantified in contralateral (contra) and ipsilateral (ipsi) sides of the spinal dorsal horns in WT and NFAT5-Het mice. Data are expressed as the means ± SEMs (one-way ANOVA with Tukey’s post-hoc test, *** *p* < 0.001 vs. WT contra, ^#^ *p* < 0.01 vs. WT ipsi; *n* = 7). (**B**) The expression levels of p-ERK in the spinal cord (lumbar [L] 4–6) were also validated by Western blot analysis. Band intensities expressed as the ratio of p-ERK normalized to total (t)-ERK. Data are expressed as the means ± SEMs (one-way ANOVA with Tukey’s post-hoc test, ** *p* < 0.05 vs. WT contra, ^##^ *p* < 0.01 vs. WT ipsi, *n* = 5). (**C**) The tissues in (A) were further co-stained with anti-*p*-ERK and anti-NeuN antibodies. Arrow heads indicate p-ERK/NeuN-positive cells. Scale bar: 50 μm (left), 20 μm (right).

In response to a diverse array of stimuli, including mitogens, osmotic stress, and proinflammatory cytokines, MAPKs are phosphorylated to direct cellular responses [32,33]. Moreover, MAPKs, such as ERK, c-Jun *n*-terminal kinase (JNK), and p38, participate in triggering pain hypersensitivity in response to inflammation [34,35]; among them, *p*-ERK is highly implicated in formalin-induced inflammatory pain [6]. Thus, we investigated the phosphorylation of ERK in response to formalin-induced inflammatory pain. We immunostained L4–6 spinal tissues from formalin-treated mice with anti-p-ERK antibodies and found that the number of p-ERK IR cells was approximately 2-fold greater on the ipsilateral side of the spinal dorsal horn compared to the contralateral side in WT mice. Interestingly, there was no significant difference in p-ERK immunoreactivity between the ipsilateral and contralateral sides of NFAT5-Het mice after formalin treatment (Figure 4A). Further, Western blot analysis of spinal dorsal horn lysates revealed that the level of p-ERK was approximately 2.3-fold higher in WT mice after formalin treatment, whereas there was no significant difference in NFAT5-Het mice (Figure 4B). To investigate the localization of p-ERK after formalin treatment, we immunostained spinal tissues with anti-p-ERK and anti-NeuN antibodies. We found that p-ERK co-localized with NeuN-positive neuronal cells in the spinal dorsal horn, and the number of p-ERK IR cells was greater in WT mice compared to NFAT5-Het mice (Figure 4C). These data indicate that formalin-induced inflammatory pain may be transmitted by the phosphorylation of ERK in neurons of the spinal dorsal horn.

### 2.5. NR2B Is Phosphorylated in Spinal Neurons in Response to Formalin-Induced Inflammatory Pain

**Figure 5:**
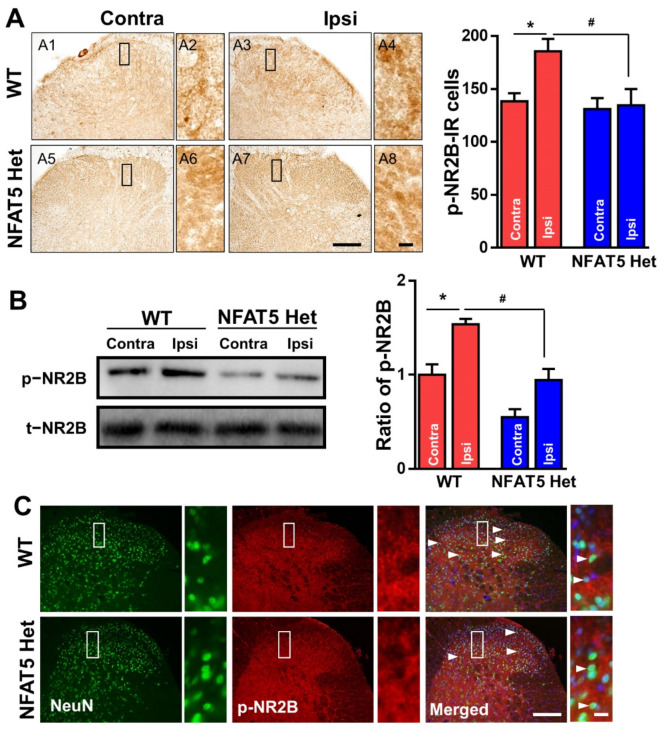
Formalin-induced phosphorylation of NR2B in the spinal dorsal horns of WT and NFAT5-Het mice. (**A**) At 1 h post-formalin injection, spinal tissues (lumbar (L) 4–6 fragments) from WT and NFAT5-Het mice were incubated with anti-p-NR2B antibody. Scale bar: 50 μm (left), 20 μm (right). The numbers of p-NR2B-immunoreactive (IR) cells were analyzed in contralateral (contra) and ipsilateral (ipsi) sides of the spinal dorsal horn in WT and NFAT5-Het mice. Data are expressed as the means ± SEMs (one-way ANOVA with Tukey’s post-hoc test, * *p* < 0.05 vs. WT contra, ^#^ *p* < 0.01 vs. WT ipsi, *n* = 5). (**B**) The expression level of p-NR2B in the spinal cord (L4–6) was also inspected by Western blot analysis. Band intensities represented as the ratio of p-NR2B normalized to total (t)-NR2B. Data are expressed as the means ± SEMs (one-way ANOVA with Tukey’s post-hoc test, * *p* < 0.05 vs. WT contra, ^#^ *p* < 0.05 vs. WT ipsi, *n* = 5). (**C**) Frozen sections from (A) above were stained with anti-p-NR2B and anti-NeuN antibodies. Arrow heads indicate p-NR2B/NeuN-positive cells. Scale bar: 50 μm (left), 20 μm (right).

*n*-methyl-D-aspartate receptor (NMDAR), a glutamate receptor, plays a pivotal role in pain by mediating excitation [36]. In the formalin-induced inflammatory pain model, NR2B, which is a subunit of NMDAR, cooperates with MAPKs to influence pain generation [6,15]. Therefore, we examined the activation of NR2B in response to formalin injection. When L4–6 spinal tissues from formalin-treated mice were immunostained with anti-p-NR2B antibodies, the number of p-NR2B IR cells was increased by approximately 33% in ipsilateral sides compared to contralateral sides of spinal dorsal horns. Interestingly, there was no significant difference between ipsilateral sides and contralateral sides of NFAT5-Het mice by formalin induction (Figure 5A). Further, in Western blot analysis of spinal dorsal horns induced by formalin, the level of p-NR2B was also increased by approximately 35% in WT mice, while there was no significant difference in NFAT5-Het mice (Figure 5B). Moreover, to investigate whether p-NR2B was phosphorylated in neurons of spinal dorsal horns by formalin treatment, the spinal tissues were immunostained with anti-p-NR2B and anti-NeuN antibodies. Consequently, p-NR2B signals were co-localized in NeuN-positive neuronal cells in dorsal horns, and the number of p-NR2B-IR cells was larger in WT mice compared to NFAT5-Het mice (Figure 5C). These data indicate that formalin-induced inflammatory pain may be transmitted by the phosphorylation of NR2B in neurons of spinal dorsal horns.

### 2.6. NFAT5 Upregulates c-Fos, p-ERK, and p-NR2B via mTOR in Glutamate-Stimulated PC12 Cells

**Figure 6:**
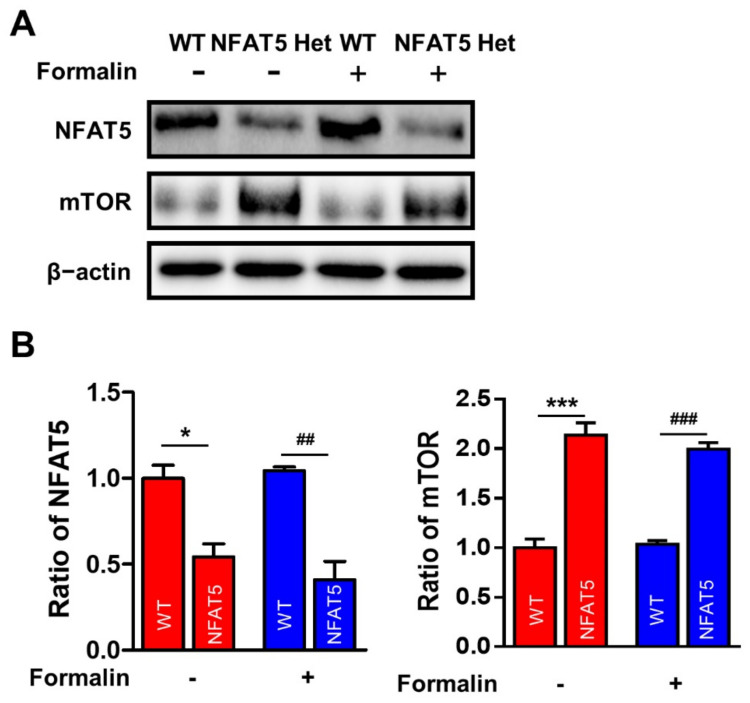
Upregulation of mTOR expression in the spinal cord of NFAT5-Het mice. (**A**) The levels of mTOR expression were examined before and after formalin injection in the ipsilateral sides of the spinal dorsal horns in WT and NFAT5-Het mice using Western blot analysis. (**B**) Band intensities represented as the ratios of mTOR normalized to β-actin. Data are expressed as the means ± SEMs (one-way ANOVA with Tukey’s post-hoc test, * *p* < 0.05 vs. WT formalin (-), *** *p* < 0.001 vs. WT formalin (-), ^##^ *p* < 0.05 vs. WT formalin (+), ^###^ *p* < 0.001 vs. WT formalin (+), *n* = 5).

As a member of the PI3K-related kinase family, mTOR regulates cell growth, proliferation, motility, and cell survival [37,38]. Furthermore, mTOR is known to influence and regulate pain generation. For example, microRNA-1906 attenuates neuropathic pain in rats by regulating the TLR4/mTOR/AKT signaling pathway [39]. Moreover, inhibition of mTOR alleviates neuropathic pain induced by the chemotherapeutic bortezomib [40], and mTOR has been identified as a potential target for chronic pain [41]. Here, we assessed the expression of mTOR in the spinal dorsal horns of WT and NFAT5-Het mice treated with or without formalin by Western blot (Figure 6). We found that the level of mTOR was approximately 2.3-fold higher in NFAT5-Het mice compared to WT mice, but mTOR expression was not further increased by formalin injection in either group.

**Figure 7:**
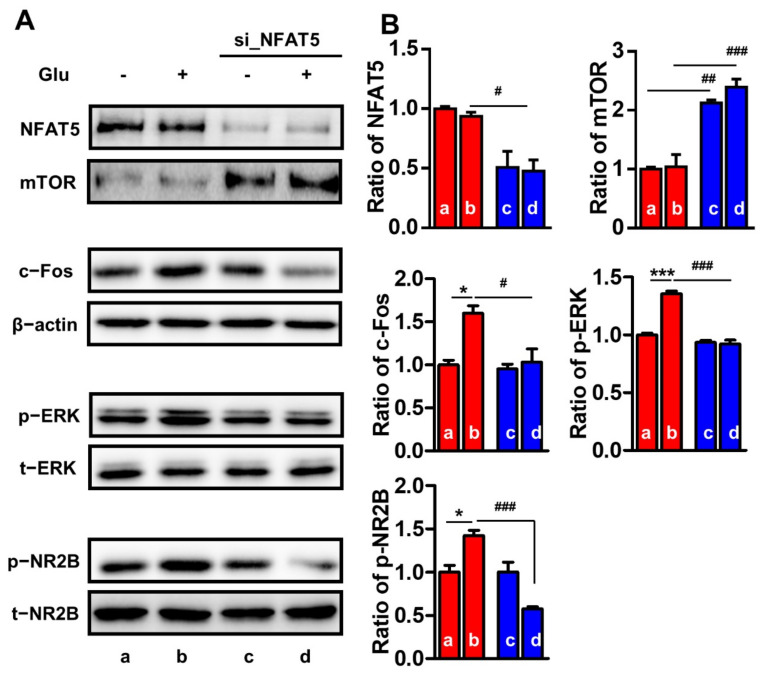
The expression of mTOR and inflammatory pain-related proteins in NFAT5-silenced PC12 cells. (**A**) The expression levels of NFAT5, mTOR, and inflammatory pain-related factors were examined using Western blot analysis in normal PC12 cells and NFAT5-silenced PC12 cells in the absence or presence of 100 μM glutamate. (**B**) Band intensities represented as the ratios of NFAT5, mTOR, and c-Fos normalized to β-actin; p-ERK normalized to total (t)-ERK; and p-NR2B normalized to t-NR2B. Data are expressed as the means ± SEMs (one-way ANOVA with Tukey’s post-hoc test, * *p* < 0.05 vs. WT formalin, *** *p* < 0.001 vs. a (negative control), ^#^ *p* < 0.05 vs. b (glutamate treatment), ^##^ *p* < 0.01 vs. b, ^###^ *p* < 0.001 vs. b, *n* = 5).

To determine whether NFAT5 and mTOR levels modulate the activation of inflammation-related proteins, such as c-Fos, p-ERK, and p-NR2B, we performed experiments in vitro. Since glutamate is highly involved in pain, inflammation, and metabolic pathways [42,43], we used glutamate as a drug to induce inflammatory-like circumstances. In response to glutamate treatment, the levels of c-Fos, p-ERK, and p-NR2B in PC12 neuronal cells increased by approximately 1.6-fold, 1.4-fold, and 1.4-fold, respectively, whereas the levels of NFAT5 and mTOR did not change (Figure 7). Further, siRNA silencing of Nfat5 in PC12 cells reduced the level of NFAT5 by approximately 50%, and the level of mTOR increased by approximately 2-fold. In contrast, the levels of the other proteins did not change. Consistent with our in vivo experiments, the levels of NFAT5, mTOR, c-Fos, p-ERK, and p-NR2B were not significantly altered by glutamate stimulation in Nfat5-silenced PC12 cells (Figure 7B). Collectively, these data imply that NFAT5 may upregulate c-Fos, p-ERK, and p-NR2B via mTOR in PC12 neuronal cells after glutamate stimulation.

## 3. Discussion

**Figure 8:**
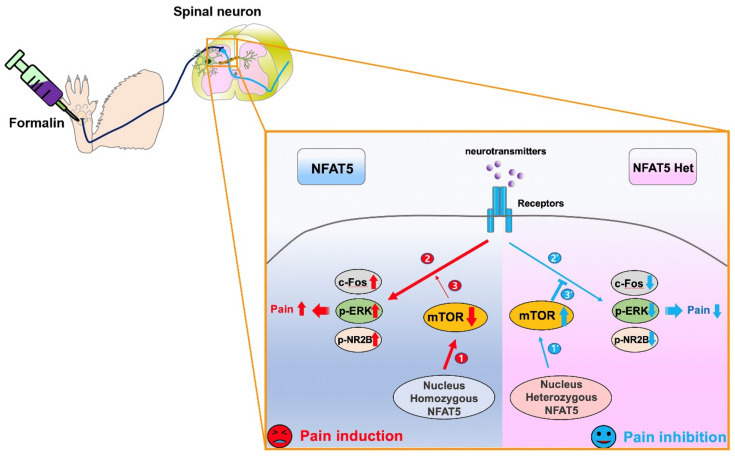
The loss of formalin-induced inflammatory pain in NFAT5-depleted mice. In normal mice, NFAT5 basically inhibits the gene expression of mTOR (**1**). When formalin was administrated to generate inflammatory pain, substances released from nerve terminals of primary afferent neurons bound to their receptors on post-synaptic neurons and induced pain by the phosphorylation of pain-related factors such as c-Fos, p-ERK, and p-NR2B in spinal neurons (2), with no interference of lower mTOR levels (3). On the other hand, in NFAT5-depleted mice, elevated levels of mTOR protein (1’) inhibited pain induction through the suppression of pain-related factors (2’, 3’).

Peripheral nociceptive inputs mediate signals in DRG neurons and activate primary afferent neurons by initiating the release of chemical mediators to the spinal cord dorsal horn [43]. Understanding these nociceptive mechanisms is key to mitigating pain, which remains clinically challenging and is often complicated by inflammation. Pain signals are amplified by the induction of inflammatory factors, which inhibit the mTOR pathway [39]. The mTOR pathway is also modulated by formalin-induced hypersensitivity and neuronal hyper-excitability [28]. Although mTOR is known to regulate pain signal transduction, the mechanisms that mediate mTOR signaling in formalin-induced inflammatory pain are still unclear. In the present study, we demonstrated that NFAT5 regulates formalin-induced inflammatory pain through the inhibition of mTOR signaling, which then regulates the expression of pain-related factors, including c-Fos, ERK, and NR2B (Figure 8).

NFAT5 contributes to inflammation and is known to respond to osmotic stress. Therefore, we hypothesized that NFAT5 may also play a role in inflammatory pain. To study inflammatory pain, we utilized a mouse model of formalin-induced acute inflammation. After unilateral formalin injection into the hind paw of NFAT5-Het and WT mice, pain behaviors were monitored and pain scores were calculated in two time-dependent phases. Once inflammatory pain was initiated via tissue damage in the second phase, NFAT5-Het mice displayed alleviated pain behaviors compared to WT mice (Figure 1). To uncover the mechanism of reduced pain in NFAT5-Het mice, we assessed the expression of proteins related to neuronal damage and nociception, including ATF3, c-Fos, ERK, NRB2, and mTOR.

We began by examining ATF3, because it is a well-known stress-inducible protein in damaged DRG neurons [30]. ATF3 expression did not change in WT or NFAT-Het mice after formalin injection, indicating that DRG neurons have minimal effects on formalin-induced inflammatory pain. This finding also suggests that the spinal dorsal horn, which is the first stop in nociceptive integration, plays a pivotal role in pain hypersensitivity after formalin injection (Figure 2). Additionally, c-Fos is expressed in the spinal dorsal horn and rapidly responds to noxious stimuli in central neurons by integrating noxious input [44]. Indeed, we observed increased expression of c-Fos and co-localization of c-Fos and NeuN in the ipsilateral sides of WT mice, confirming that neurons in the spinal dorsal horn are activated by nociceptive input after formalin injection [45,46]. In contrast, NFAT5-Het mice had low levels of c-Fos on both sides of the spinal dorsal horn compared to WT mice and reduced co-immunostaining of c-Fos and NeuN (Figure 3). Similar to c-Fos, both ERK and NR2B are downstream of mTOR. As ERK and NR2B are also considered initiation factors of inflammatory pain, we examined their expression in the spinal dorsal horn. NFAT5-Het mice showed diminished immunoreactivity and protein levels of p-ERK and NR2B compared to WT mice. Moreover, co-localization of p-ERK or NR2B with NeuN was notably reduced (Figure 4 and Figure 5, respectively). These results mirrored those of c-Fos and suggest that NFAT contributes to the regulation of these pain-related factors through a common mechanism, likely via mTOR signaling.

To uncover the regulation of pain-related factors, we investigated the effects of NFAT5 on the expression of mTOR during the generation of formalin-induced inflammatory pain. It is widely reported that mTOR participates in the regulation of chronic pain, and a recent report showed that NFAT5 inhibits mTOR and notch signaling [47]. Here, we demonstrated that mTOR expression was elevated in NFAT5-Het mice regardless of formalin injection (Figure 6), confirming that mTOR is downregulated by NFAT5. To further study and validate this mechanism, we used the well-known PC12 neuronal cell line. Consistent with our in vivo studies, pain-related factors diminished in Nfat5-silenced PC12 cells compared to negative controls (Figure 7).

Taken together, these results demonstrate that inhibition of NFAT5 regulates pain-related factors by upregulating the expression of mTOR and downregulating its downstream factors. Based on these results, we suggest that NFAT5-selective inhibitors, such as KRN2 and KRN5, could alleviate formalin-induced inflammatory pain by upregulating the expression of mTOR and downregulating its downstream factors.

## 4. Materials and Methods

### 4.1. Animals

C57BL/6 mice (8-week-old, male) were obtained from Damul Bioscience, Inc. (Daejeon Chungnam, Korea). NFAT5-Het mice were kindly provided by Dr. H. M. Kwon (UNIST, Ulsan, Korea). All mice were housed in cages under standard conditions (23 ± 2 °C, 50% humidity, and a 12 h light/dark cycle) with water and food provided ad libitum. All animal experiments were performed with the approval of the Animal Care and Use Committee of Chungnam National University (CNU-01075, 15 June 2018) and were consistent with the ethical guidelines of the National Institutes of Health and the International Association for the Study of Pain [48].

### 4.2. Formalin Test

The formalin test was carried out as described previously [6]. In brief, 10 µL of 2% formalin (Sigma-Aldrich, St. Louis, MO, USA) was administered under the skin of the plantar surface of the right hind paw of mice using a 26.5-gauge needle. Formalin-induced pain behaviors, such as licking, lifting, and flinching, were observed for 60 min at 5 min intervals in individual cages. For analyses, pain scores were summed and grouped into two phases: phase I (0–15 min) and phase II (15–60 min). All tests were performed between 11:00 and 15:00 in a quiet room. After behavioral testing, spinal tissues and DRG samples were isolated for immunohistochemistry and Western blotting as detailed below.

### 4.3. Tissue Processing and Immunostaining

Mice were deeply anesthetized with an intraperitoneal injection of 100 mg/kg sodium pentobarbital (Sigma-Aldrich, St. Louis, MO, USA) and then perfused with 2% heparinized phosphate-buffered saline (PBS, pH 7.4) and ice-cold 4% paraformaldehyde (Biosesang, Sungnam, Republic of Korea) in PBS using a peristaltic pump at a rate of 20 mL/min. DRG and lumbar (L4–6) fragments of the spinal cord were immediately isolated and post-fixed in the same fixative overnight at 4 °C. After 48 h, tissues were immersed in a series of sucrose solutions (Sigma-Aldrich, St. Louis, MO, USA) in PBS (from 10–30%) for cryopreservation. After 2 days, tissues were mounted in optimal cutting temperature (OCT) compound (Sakura Finetek, Nakano, Japan) sectioned at 30 µm using a cryostat microtome (CM1950; Leica, Wetzlar, Germany), and stored at −20 °C in storage buffer with 30% glycerol (Merck Millipore, MA, USA) 30% ethylene glycol (Merck Millipore, MA, USA) in PBS or at −70 °C in a deep freezer [49].

For diaminobenzidine (DAB) staining, spinal sections were incubated in 1% hydrogen peroxide (H2O2, Ducksan science, Seoul, Republic of Korea) in PBS for 10 min to block endogenous peroxidase activity and were further incubated with blocking buffer, 3% triton X 100 (Sigma-Aldrich, St. Louis, MO, USA), 5% chicken serum (Sigma-Aldrich, St. Louis, MO, USA) in PBS for 1 h at room temperature. The sections were then treated with a primary antibody (c-Fos, 1:400, Abcam, Cambridge, UK, ab190289; p-ERK, 1:400, Cell Signaling Technology, Danvers, MA, USA, 9101; p-NR2B, 1:400, Cell Signaling Technology, 4370T) overnight at 4 °C, followed by a biotinylated secondary antibody and a streptavidin peroxidase complex (Vector Laboratories, Inc., Burlingame, CA, USA). The specimens were visualized with DAB–peroxidase substrate solution, 0.05% DAB (Thermo Fisher Scientific, Waltham, MA, USA)/0.015% H2O2 (Ducksan Science, Seoul, Republic of Korea) in PBS and mounted on glass slides using Polymount solution (Polysciences, Warrington, PA, USA). Images were obtained using brightfield microscopy (ECLIPSE E600 POL; Nikon, Tokyo, Japan).

For fluorescent staining, tissues were first exposed to blocking buffer (Thermo Fisher Scientific, Waltham, MA, USA) for 1 h at room temperature. The tissues were then incubated with a mixture of primary antibodies (c-Fos, 1:400, Abcam, ab190289; p-ERK, 1:400, Cell Signaling Technology, 9101; p-NR2B, 1:400, Cell Signaling Technology, 4370T; NeuN, 1:400, Cell Signaling Technology, 324307) diluted in blocking buffer overnight at 4 °C, followed by a mixture of corresponding secondary antibodies conjugated with either FITC or Cy3 (1:400; Jackson ImmunoResearch, West Grove, PA, USA) diluted in blocking buffer [50]. The tissues were counterstained with DAPI (5 g/mL in PBS; Thermo Fisher Scientific, Waltham, MA, USA) for 5 min and then mounted on glass slides with mounting medium (Biomeda, Foster City, CA, USA). Images were obtained using a laser scanning microscope (TCS SP8; Leica) and used for additional data analysis.

### 4.4. Western Blot Analysis

L4–6 fragments of the ipsilateral spinal dorsal horn post-PBS perfusion were rapidly isolated and homogenized in protein lysis buffer (PRO-PREP; Intron, Daejeon, Republic of Korea). Following the quantitation of cell lysates with the Bradford assay (Bio-Rad, Hercules, CA, USA), 20 µg of each lysate was separated on 8 or 10% SDS-polyacrylamide gels and transferred on polyvinylidene fluoride (PVDF) membranes (Millipore, Burlington, MA, USA). After blocking with 5% non-fat skim milk in Tris-buffered saline (pH 7.4) supplemented with 0.3% Tween-20 for 1 h at room temperature, the blots were probed with primary antibodies (c-Fos, 1:1000, Abcam, ab190289; p-ERK, 1:1000, Cell Signaling Technology, 9101; t-ERK, 1:1000, Cell Signaling Technology, 9102; p-NR2B, 1:1000, Abcam, ab81271; t-NR2B, 1:1000, Millipore, 06-600; β-actin, 1:2000, Cell Signaling Technology, 2965). Membranes were then incubated with secondary antibodies conjugated with horseradish peroxidase (HRP; anti-rabbit-HRP or anti-mouse-HRP; KOMA, Seoul, Korea). Bands were detected and imaged using the ChemiDoc Touch imaging system (Habersham, Little Chalfont, UK) with an enhanced chemiluminescence solution (GE Healthcare, Little Chalfont, UK). All experiments were performed in triplicate. The band intensities were measured using Image J (NIH, Bethesda, MD, USA), normalized with ß-actin or total protein, and represented as a ratio.

### 4.5. Cell Culture and Nfat5 siRNA Transfection

The PC12 rat neuronal cell line was purchased from the Korea Cell Line Bank (KCLB, Seoul, Korea) and maintained in minimum essential medium (Welgene Biotech, Kyungsan, Korea) supplemented with 10% heat-inactivated fetal bovine serum (Thermo Fisher Scientific, Waltham, MA, USA) and 1% antibiotics (Sigma-Aldrich, St. Louis, MO, USA) in a humidified CO2 incubator at 37 °C. PC12 cells were seeded at 5 × 10^5^ cells/well in 6-well plates. The following day, cells were transiently transfected with negative control Hi GC siRNA (20 nM; Thermo Fisher Scientific) or Nfat5 siRNA (20 nM; Thermo Fisher Scientific) using Lipofectamine RNAiMAX (Thermo Fisher Scientific) according to the manufacturer’s instructions. After 48 h, the cells were used in experiments. For some experiments, cells were treated with 100 μM glutamate for 1 h after Nfat5 silencing.

### 4.6. Statistical Analysis

Data are expressed as the means ± standard errors of the mean (SEMs). Differences between multiple groups were determined by one- or two-way analysis of variance (ANOVA), followed by a Tukey’s test. *p*-values < 0.05 were considered statistically significant. All statistical analyses were performed using GraphPad Prism 6 (GraphPad Software Inc., La Jolla, CA, USA).

## Data Availability

All data generated or analyzed during this study are included in this published article.
